# Integrated Heat Recovery System Based on Mixed Ionic–Electronic Conducting Membrane for Improved Solid Oxide Co-Electrolyzer Performance

**DOI:** 10.3390/polym16070932

**Published:** 2024-03-28

**Authors:** José Sánchez-Luján, Ángel Molina-García, José Javier López-Cascales

**Affiliations:** 1 Department of Chemical and Environmental Engineering, Universidad Politécnica de Cartagena, 30203 Cartagena, Spain; javier.lopez@upct.es; 2 Department of Automatics, Electrical Engineering and Electronic Technology, Universidad Politécnica de Cartagena, 30202 Cartagena, Spain; angel.molina@upct.es

**Keywords:** membrane, MIEC, oxygen separator, solid oxide co-electrolyzer

## Abstract

The current state of mixed ionic–electronic conducting ceramic membrane technology presents significant advancements with potential applications in various fields including solid oxide electrolyzers, fuel cells, hydrogen production, CO_2_ reduction, and membrane reactors for chemical production and oxygen separation. Particularly in oxygen separation applications, optimal conditions closely align with the conditions of oxygen-rich air streams emitted from the anode of solid oxide co-electrolyzers. This paper describes and analyzes a novel integrated heat recovery system based on mixed ionic–electronic conducting membranes. The system operates in two stages: firstly, oxygen is separated from the anode output stream using mixed ionic–electronic conducting membranes aided by a vacuum system, followed by the heat recovery process. Upon oxygen separation, the swept gas stream is recirculated at temperatures near thermoneutral conditions, resulting in performance improvements at both cell and system levels. Additionally, an oxygen stream is generated for various applications. An Aspen HYSYS^®^ model has been developed to calculate heat and material balances, demonstrating the efficiency enhancements of the proposed system configuration.

## 1. Introduction

In addition to the implementation of energy transition policies within the European Union (EU) [1,2] to facilitate a shift towards sustainable energy production, distribution, and consumption models, the ongoing conflict in Ukraine has underscored the EU’s external energy dependency [3,4]. Under this framework, innovative technologies aimed at promoting efficient energy utilization and reducing reliance on fossil fuels are of paramount importance. Among these emerging technologies, solid oxide electrolyzers (SOEC) [5] and mixed ionic–electronic conducting (MIEC) membranes [6] have emerged as promising solutions for converting renewable energy into hydrogen and syngas [7]. These technologies offer potential pathways for both advancing energy transition objectives and enhancing energy independence within the European Union [8,9,10].

The current technological development of MIEC membranes allows their use in applications such as H_2_ production, O_2_ separation, CO_2_ reduction, membrane reactors, and material development for fuel cell cathodes and solid oxide electrolyzers for synthetic fuel processes [11,12]. Numerous contributions and studies have focused on the production of syngas via co-electrolysis to generate synthetic fuels. Hänggi et al. [13] presented a comprehensive review of synthetic fuels for passenger vehicles, providing insights into the potential of such fuels. Singh et al. [14] emphasized the sustainability of hydrogen as a fuel for the future of the transport sector. In their work, Wilhelm et al. [15] explored syngas production for gas-to-liquid applications, addressing various technologies, issues, and future outlooks. Wang et al. [16] conducted an optimal design study of solid oxide electrolyzers for power-to-methane systems, comparing steam electrolysis and co-electrolysis. Santos and Alencar [17] reviewed biomass-derived syngas production through the gasification process, offering a catalytic conversion perspective via Fischer–Tropsch synthesis. Liu et al. [18] investigated the electrochemical generation of syngas from water and carbon dioxide at industrially significant rates. Additionally, Yang et al. [19] explored recent advances in co-thermochemical conversions of biomass with fossil fuels, focusing on synergistic effects. Delgado Dobladez et al. [20] proposed an efficient recovery method for syngas from dry methane reforming products using a dual pressure swing adsorption process. Gupta et al. [21] discussed renewable fuels through Fischer–Tropsch synthesis, while Becker et al. [10] explored the production of Fischer–Tropsch liquid fuels using high-temperature solid oxide co-electrolysis units. Herz et al. [22], Tremel et al. [23], König et al. [24], Wang et al. [25], Luo et al. [26], and Liu et al. [18] contributed to the techno-economic analysis, simulation, and evaluation of various processes related to synthetic fuel generation and hydrogen production. These studies collectively enrich the understanding of syngas production technologies and their potential applications.

With regard to the oxygen production and modeling by MIEC membranes, a variety of contributions can be also found in the specific literature. Schiestel et al. [27] explored the application of hollow fiber perovskite membranes for oxygen separation, providing valuable insights into rapid communication in membrane science. Lia et al. [28] presented an overview of oxygen transport through MIEC ceramic-based membranes, offering a comprehensive modeling perspective. Sunarso et al. [29] delved into the development of MIEC ceramic-based membranes for oxygen separation, contributing to the understanding of their properties and applications. Jin et al. [30] focused on the sequential simulation of dense oxygen permeation membrane reactors for hydrogen production, incorporating Aspen Plus in their study. Kriegel [31] investigated the competitiveness of MIEC membrane plants for commercial oxygen production, providing a unique perspective on their potential. Chen et al. [11] presented a roadmap for sustainable mixed ionic–electronic conducting membranes, contributing to the advancement of membrane technologies. Catalán-Martínez et al. [32] characterized oxygen transport phenomena on Ba_0.5_Sr_0.5_Co_0.8_Fe_0.2_O_3−δ_ (BSCF) membranes through fluid dynamic simulations, expanding the understanding of surface exchange. Khajryan et al. [33] explored O_2_ separation using MIEC membranes and a steam circulation process, demonstrating its potential application. Arratibel Plazaola et al. [34] provided a comprehensive review of mixed ionic–electronic conducting membranes (MIEC) for their application in membrane reactors, consolidating knowledge in the field. Engels S. et al. [35] simulated a membrane unit for oxyfuel plants in Aspen, calibrating the model with experimental data obtained from laboratory testing of a tubular BSCF membrane. Spallina et al. [36] modeled the autothermal reforming of methane in a BSCF tubular membrane reactor with co-current flow using the Xu–Thomson model [37]. Fischer and Iribarren [38] used Aspen Custom Modeler to simulate a BSCF membrane in three-end and four-end modes with counter-current flow. Turi et al. [39] employed Aspen Custom Modeler for a La_0.9_Ca_0.1_Fe_3−δ_ planar membrane with porous supports. They utilized the Xu–Thomson model [37] to calculate the permeation flux, while the van Hassel model [40] was employed to calculate the mass transfer through a porous support. The practical applications of MIEC membranes continue to face numerous challenges, such as long-term stability, improvement of mechanical strength, and low chemical stability [41,42,43]. An alternative to perovskite is the monophasic K_2_NiF_4_-type membrane (Ruddlesden–Popper phase), which has proven to be completely CO_2_-tolerant during long-term stability tests in a CO_2_ atmosphere. Similarly, biphasic membranes have also demonstrated high CO_2_ resistance after prolonged exposure without carbonate formation or phase changes. Although CO_2_ tolerance has significantly improved in K_2_NiF_4_-type and dual-phase membranes, their oxygen permeability is much lower compared to perovskite-type oxygen transport membranes (OTMs). The apparent trade-off between oxygen permeation flux and CO_2_ tolerance limits the applicability of OTMs for oxygen separation [44,45].

**Figure 1:**
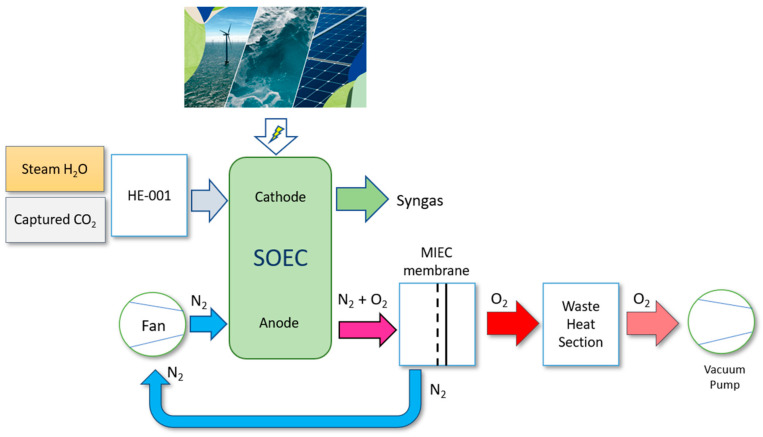
Block diagram of the integration of the oxygen separation system and heat recovery from the anode sweep gas stream.

By considering previous contributions, this paper analyzes a heat recovery system in the anode outlet stream of a co-electrolyzer producing syngas from captured CO_2_, steam, and renewable electricity [9]. The anode outlet stream consists of oxygen-enriched sweep air generated during co-electrolysis, in water and CO_2_ reduction reactions, with a temperature of 850 °C. Typically, the heat from this stream is recovered to usable levels in other process and auxiliary streams [5]. The novelty and main contributions of the present paper, based on using MIEC membranes, consist of two objectives: (i) the full utilization of available heat in this stream; and (ii) the extraction of oxygen by vacuum, allowing its use in subsequent industrial applications and processes [13,14,15,16,17]. Once oxygen is separated from the anode sweep air stream, the sweep air can be recirculated with the necessary inlet conditions to maintain thermoneutral operation at the anode. Furthermore, the objective is to thermally integrate the extracted oxygen stream with the cathode feed, preheated with high-pressure steam generated by the cathode syngas outlet stream, and thus providing the necessary heat for thermoneutral co-electrolyzer operation [5]. A series of improvements in the co-electrolyzer of a synthetic fuel production plant were proposed by the authors in previous works [46]. The novel approach lies in the incorporation of an integrated heat recovery system and an oxygen separation system using MIEC membranes. Theoretically, perovskite-type MIEC ceramic membranes can separate oxygen with 100% selectivity, offering an efficient and simplified method for O_2_ production. The MIEC membrane-based separation method can reduce energy consumption by 60% and significantly cut production costs by approximately 35% compared to current cryogenic technology [11]. Figure 1 illustrates a diagram of the integrated heat recovery and oxygen extraction system.

The rest of the paper is structured as follows: Section 2 describes the theoretical background and the corresponding model selection. Section 3 demonstrates the modeling technology. Results and discussion are provided in Section 4. Finally, conclusions are given in Section 5.

## 2. Theoretical Background and Model Selection

### 2.1. Modeling MIEC Membranes for Oxygen Separation

**Figure 2:**
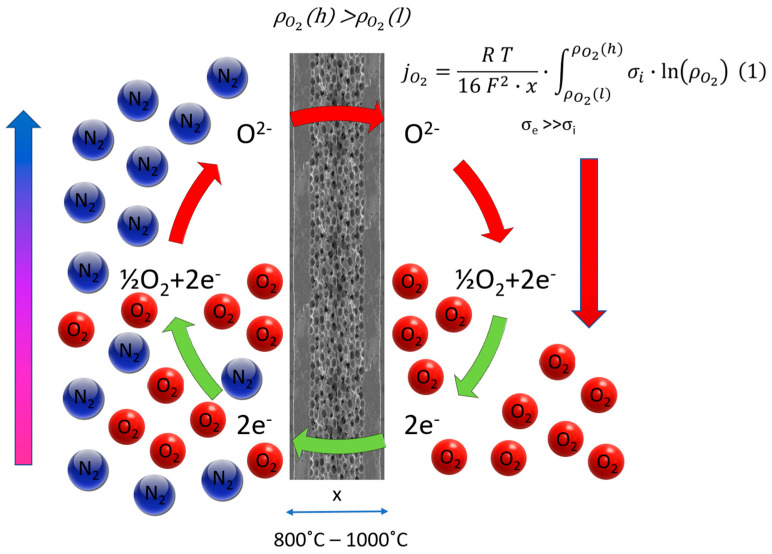
Oxygen separation mechanism and surface exchange reactions. Wagner equation.

The oxygen permeation through mixed conducting membranes is typically governed by two primary factors: bulk transport within the membrane material and surface kinetics on both sides of the membrane. In the case of a thick membrane, the Wagner equation in Figure 2 describes the oxygen permeation flux, with solid state diffusion dominating and being influenced by the ratio of electron conductivity (σ_el_) to ion conductivity (σ_ion_).

The Wagner Equation (1) is simplified using an Arrhenius approach:

(1) $$j_{O_{2}}=\frac{RT}{16F^{2}\cdot x}\cdot \int_{\rho_{O_{2}}(l)}^{\rho_{O_{2}}(h)}\sigma_{i}\cdot \ln\left(\rho_{O_{2}}\right)$$

(2) jO2=C Tmemdmeme−KTmemln⁡ρO2,f/rρO2,s/p 

where *C* and *K* are material constants, *T_mem_* is the membrane temperature, and $\rho_{O_{2}}$ is the oxygen partial pressure. Oxygen permeation in dense MIEC membranes occurs through ionic and electronic transfers, facilitated by defects like oxygen vacancies and interstitial oxygen ions within the crystal lattice. The defects enhance oxygen ion diffusion, contributing to ionic conductivity. Oxygen permeation models for MIEC membranes consider the transport of ionic and electronic species through the membrane, with the Wagner theory [47] representing an early model based on bulk diffusion. The determined values for *C* and *K*, fitted by Engels S. et al. [35], are 1.004 × 10^−8^ (mol/cm sK) and 6201 K, respectively. Figure 2 illustrates the excellent match between the fitted Wagner equation and the experimental data within the temperature range of 750–900 °C. The experimental investigations confirm the validity of the logarithmic function of the oxygen partial pressure ratio in the Wagner equation. This validity remains independent of absolute pressures on the feed and permeate sides within the applied pressure range. Consequently, the fitted Wagner equation is deemed suitable for simulations in both three- and four-end membrane module concepts.

Surface exchange reactions play a crucial role in oxygen permeation in MIEC membranes, occurring between molecular oxygen and oxygen vacancies on the membrane interfaces. The slowest step, whether surface exchange reactions or bulk diffusion, limits oxygen permeation. Empirical equations have been developed to predict oxygen permeation fluxes for various limiting cases. Surface exchange reactions involve molecular oxygen incorporation, lattice oxygen diffusion, and electron flow.

(3) $$h=\frac{k}{D^{*}}=\frac{1}{L_{c}}$$

(4) jO2=krkf−P″O2−1/2−P’O2−1/21KfP’O21/2+2LDv+1KfP″O21/2,

where *k_f_* and *k_r_* represent forward and reverse surface exchange reaction rates, respectively. This expression incorporates *k_f_* and *k_r_* for membrane feed and permeate sides, providing the interplay of these reactions. The ratio of surface exchange kinetics (*k*) to tracer diffusion coefficient (D*) is defined as the h-parameter, giving insights into the influence of surface exchange reactions on oxygen permeation (see Equation (3)). The characteristic thickness, *L_c_*, introduced by Bouwmeester et al. [48] is the inverse of the h-parameter and allows determination of whether oxygen permeation is limited by bulk diffusion or surface exchange reactions based on membrane thickness. This analysis provides a comprehensive understanding of the mechanisms governing oxygen permeation in MIEC membranes and lays the groundwork for further research in this domain.

Several models, including the Tan and Li model [49], and Ghadimi model [50], have been developed based on the assumptions of the Xu–Thomson model [37], with incremental modifications aimed at enhancing applicability. The Xu–Thomson model, initially founded on Lin et al.’s surface reaction model, serves as the foundational framework; this model for the oxygen permeation flux, as shown in Equation (4), assumes a constant oxygen vacancy diffusion coefficient *D_v_*, considering ideal gas in steady-state isothermal. In contrast, the Zhu model [51] adopts a different approach, deriving from the chemical potentials of the transported species in the membrane. Alternative modeling approaches include the effective medium approximation and theories such as the Wagner theory, percolation theory, and defect chemistry. Following a comprehensive study and evaluation of the existing models for oxygen permeation in MIEC membranes, the Tan and Li model [49] emerges as the most suitable for the selected materials, geometry, and process conditions. This model, developed as a modification of the Xu–Thomson model, is specifically tailored for tube membrane geometries, encompassing tubular and hollow fiber membranes, including U-shaped hollow fibers. The model expresses the local differential oxygen molar flow rate over a differential membrane length, effectively addressing the oxygen partial pressure variation along the membrane length. The Tan and Li model [49] has been successfully applied to simulate perovskite (La_0.6_Sr_0.4_Co_0.2_Fe_0.8_O_3−δ_ and BSCF) and surface-activated dual-phase membranes. Commercial process simulation software like Aspen Plus^®^ V14 proves useful for simulating hybrid processes incorporating membrane units. However, standard Aspen Plus lacks modules for membrane processes, requiring users to create membrane models in Fortran, Microsoft Excel, or JACOBIAN code and then import them into Aspen Plus. Alternatively, Aspen Custom Modeler, a product within the Aspen Plus suite, allows the creation of rigorous process equipment models. Once integrated into Aspen Plus, the membrane model functions like any other model in the library. The flexibility of Aspen Plus enables the simulation of reactions occurring in membranes integrated with entire systems. A detailed description of the process model is provided in Section 3.1, elaborating on the models used for characterizing and simulating the membrane behavior.

### 2.2. Oxygen Separation System Based on MIEC Membranes

In the present paper, and for intended application, optimal conditions for separation with MIEC membranes are described. This proposal focuses on removing the need for additional thermal energy to heat the gas stream. This advantageous scenario allows for a more efficient and streamlined separation process. Evaluations of different possible system configurations lead to a system based on vacuum capillaries and three-end mode operation. The capillary geometry offers a remarkable specific surface area, making it highly effective for separation processes. In comparison to conventional geometries such as discs, tubes, and multichannel elements, membranes based on capillaries boast the highest packing density (separation area per unit volume) and require an extremely low material consumption [52,53]. In the state-of-the-art product development and solutions at Fraunhofer IKTS, stiff-plastic extrusion techniques are employed to create monolithic tubes and capillaries. Ongoing investigations focus on advancing membrane technology, aiming for higher oxygen flux and improved packaging density. This involves exploring asymmetric designs that incorporate a thin separation layer on a porous support structure. Additionally, efforts are directed toward the development of multichannel tubes and bunches of capillaries, along with combinations of these innovative approaches, including coatings for high-flux, oxygen-permeable membranes. These advancements signify a continuous pursuit of efficiency and performance in membrane-based separation systems [31,53]. In this case, the approach is based on the latest advancements and solutions proposed by Fraunhofer ITKS in the Basics of MIEC Membrane Plants for O_2_ Production [31,44].

The simplest gas management method for O_2_ production involves a crossflow three-end arrangement.

**Figure 3:**
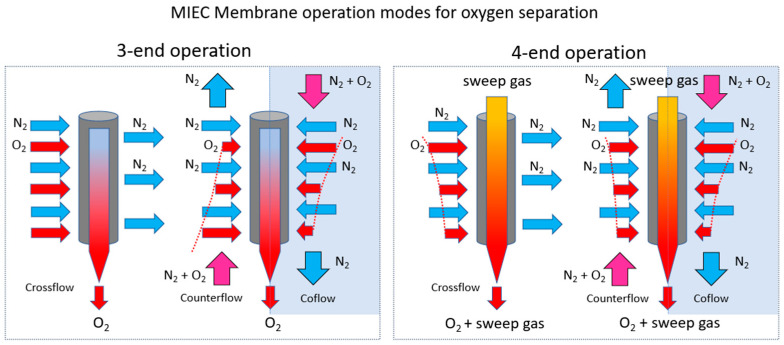
MIEC membrane operation modes for oxygen separation.

Figure 3 summarizes the MIEC membrane operation modes for oxygen separation purposes. MIEC membranes can operate with or without a sweep gas, known as four-end and three-end operations, respectively [54,55]. The former represents a membrane reactor, while the latter is a membrane separator. Gas management is more challenging for a membrane reactor, and material requirements are typically higher. In the anode of the co-electrolyzer, N_2_ as a sweep gas will be implemented within the four-end configuration of the solid oxide electrolysis cell (SOEC) bringing several advantages [56,57]. The use of a sweep gas (in this case, N_2)_ facilitates improved cell performance and efficiency. It serves to remove any excess water vapor and oxygen from the anode compartment, preventing their accumulation and enhancing the overall electrochemical reactions. The advantages associated of four-end mode, N_2_ sweep gas, in the anode of the co-electrolyzer are stated below.

- Reduced overpotential: The presence of N_2_ as a sweep gas helps in maintaining optimal operating conditions, minimizing overpotential at the anode. This, in turn, improves the kinetics of the electrochemical processes, leading to enhanced overall efficiency.
- Enhanced reaction rates: The removal of water vapor and oxygen through the sweep gas prevents side reactions and corrosion, contributing to more controlled and efficient electrochemical reactions at the anode. This results in improved reaction rates and electrolyzer performance.
- Extended cell lifespan: By preventing the accumulation of detrimental by-products, such as carbon deposition and oxide formation, the utilization of N_2_ as a sweep gas can contribute to an extended lifespan of the SOEC. This is crucial for the long-term durability and reliability of the co-electrolyzer.
- Improved gas purity: The presence of a sweep gas helps in maintaining a cleaner anode environment by carrying away impurities. This ensures the production of high-purity hydrogen or other desired products at the cathode without contamination from unwanted gases.
- Optimized gas distribution: The controlled flow of N_2_ as a sweep gas aids in achieving a well-distributed gas gradient within the anode compartment. This is essential for uniform electrochemical reactions across the electrode surface, preventing localized issues and ensuring consistent performance.

In conclusion, the implementation of N_2_ as a sweep gas in the anode of the co-electrolyzer operating in a four-end SOEC configuration offers multiple benefits, ranging from improved efficiency and reaction rates to extended cell lifespan and optimized gas purity. Achieving a well-defined gas distribution at high temperatures, such as a counterflow of gases, is difficult and expensive in terms of construction details. Moreover, membrane reactors often require gas-tight joints or sealings of ceramic membranes to special steel alloys suitable for high temperatures. These joints pose a safety risk, as do the brittle membranes separating highly flammable gas from glowing air. Additionally, technical plants often demand high gas pressures to meet the requirements of subsequent process steps. In cases of significant pressure differences, tubular MIEC membranes are preferred over planar ones.

## 3. Methodology Modeling

### 3.1. Process Model General Description

The MIEC integration for oxygen separation from the outlet stream of the anode of the SOEC co-electrolyzer, which involves oxygen-enriched sweep nitrogen generated during co-electrolysis, in the water and CO_2_ reduction reactions, at a temperature of 850 °C, allows the unit to continuously supply oxygen to other industrial processes or equipment such as the catalytic partial oxidation reactor (CPOX). This can be coupled with the co-electrolyzer for the modulation of uninterrupted syngas production. The various membrane materials and typologies, as well as the optimal operating conditions for designing the oxygen separation system using membranes and the heat recovery system within the process, have been analyzed. Furthermore, an integration of the heat recovery system was conducted to assess the enhancement of efficiency at the co-electrolyzer level. The application of MIEC membranes for oxygen separation aims to achieve the following objectives:

- Valorization of the extracted oxygen through vacuum in other industrial applications and processes.
- Recovery of the available heat in the oxygen stream.
- Energy savings at the co-electrolyzer system level by being able to recirculate the sweep gas stream, avoiding the need to provide the heat required to reach the temperature of the thermoneutral mode from ambient temperature.

**Figure 4:**
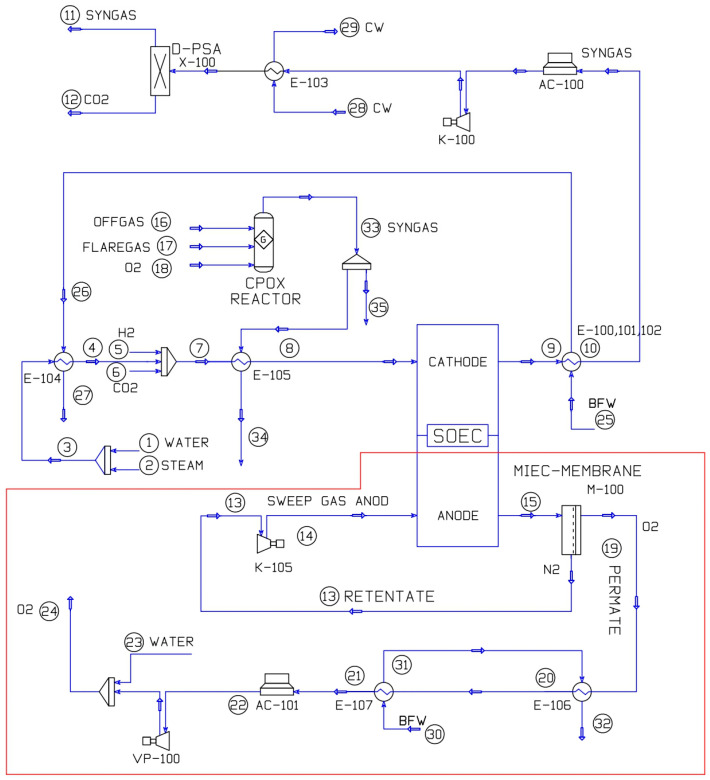
Flow sheet MIEC membrane and heat recovery system integration details.

This section is thus focused on the description in the part of the flow sheet related to the oxygen separation MIEC membrane and the heat recovery system. In the proposed process model, developed in Aspen HYSYS^®^ software V14 (Bedford, MA, USA), an oxygen separation system has been integrated at the anode outlet of the co-electrolyzer, using bundles of capillaries with MIEC membranes, BSCF material, operating in a three-end vacuum configuration. This setup results in an oxygen stream in the permeate and a nitrogen-rich retentate with residual oxygen content. The retentate stream, maintaining the conditions of the co-electrolyzer anode outlet, would be recirculated as the anode sweep gas, resulting in energy savings from preheating and conditioning this stream to keep the co-electrolyzer at the optimum thermoneutral operating point. The permeate stream passes through a heat recovery system where steam is used as the carrier. The system consists of two main equipment: a process gas boiler (PGB), labeled as E-101, and an evaporator, labeled as E-102. This steam-based heat recovery system is integrated at the system level within the co-electrolyzer, contributing part of the steam required for co-electrolysis. After exiting the heat recovery system, the oxygen stream is cooled using an air cooler. Subsequently, through a tempering or superheating process involving water spraying via a nozzle into the oxygen stream, the oxygen is further conditioned. The condensation of water allows for the easy production of pure but wet oxygen, and the thermal energy required for water evaporation and recondensation is provided. Finally, a chiller is employed to achieve a temperature compatible with the optimal operating conditions of the multi-stage vacuum system (see Table 1). Figure 4 depicts the flow sheet of the process model, highlighting the novel contribution. The rest of the flow sheet is explained in a previous work of Sánchez-Luján et al. [46].

Although the standard Aspen Plus^®^ V14 software lacks modules for membrane processes, users can create membrane models in Fortran, Microsoft Excel, etc. and import them into Aspen Plus^®^ or develop them through Aspen Custom Modeler. The development of a model for MIEC (mixed ionic–electronic conducting) membranes in Aspen Custom Modeler involved a specialized approach, defining the type of MIEC membrane composition, structure, and key properties. Relevant model components, including ionic and electronic transport species and any other pertinent parameters, were selected. The Wagner theory [47] was incorporated into the model by implementing equations that describe the oxygen permeation process through MIEC membranes based on Wagner’s principles. The bulk diffusion was considered as the primary transport mechanism for oxygen within the membrane, following the simplified form of the Wagner theory derived from Equation (1). The Wagner theory posits a local equilibrium between charged species (oxygen ion and electron) and a hypothetical neutral species (such as oxygen molecules) in the bulk oxide. The oxygen permeation flux can be estimated as

(5) $$\;J_{O_{2}}=\frac{1}{4^{2}F^{2}L}\int_{\mu_{O_{2}}(II)}^{\mu_{O_{2}}(I)}t_{ion}t_{e}\sigma^{tot}d\mu_{O_{2}}\;$$

where *L* is the disk membrane thickness (cm), *t_ion_* is the oxygen ionic transfer number, *t_e_* is the oxygen electronic transfer number, *σ^tot^* is the total conductivity (S/cm), and $\mu_{O_{2}}$ is the chemical potential of the hypothetical neutral oxygen in the oxide (J/mol).

The oxygen ionic transfer number, *t_ion_*, correlates with membrane conductivity through the following expression:

(6) $$t_{ion}=\frac{\sigma_{ion}}{\sigma_{ion}+\sigma_{e}}$$

where *σ_ion_* is the ionic conductivity (S/cm) and *σ_e_* is the electronic conductivity (S/cm).

The Tan and Li model [49] was integrated into the membrane model, addressing specific aspects related to MIEC membrane behavior such as surface reactions, diffusion, and electrochemical processes. The process flow within the MIEC membrane model was defined, considering the flow of ionic and electronic species, reactions at the membrane interfaces, and relevant transport phenomena. Transport phenomena specific to MIEC membranes, including ionic and electronic conductivity, surface exchange reactions, and any other relevant processes described by the Tan and Li model, were addressed. Boundary conditions were defined based on the Wagner theory and the Tan and Li model, reflecting the operating environment, temperature, pressure, and concentration gradients.

Under Aspen^®^ modeling conditions, boundary conditions are crucial [30,58,59,60]. For the three-end concept, the membrane’s performance depends on permeate pressure, feed temperature and pressure, and the oxygen separation ratio. The four-end concept introduces additional parameters like the oxygen concentration of the sweep stream and its temperature. These parameters influence the oxygen permeation rate and, consequently, the required membrane area. Tan and Li [49] expanded the Xu–Thomson model for hollow fiber membranes, introducing several assumptions tailored to this configuration. Similar to the Xu–Thomson model [37], assumptions regarding axial diffusion, negligible gas-phase mass transfer resistances, and plug-flow conditions were maintained. Specific assumptions for the Tan and Li model included neglecting axial diffusion due to the long hollow fiber length, assuming negligible gas-phase mass transfer resistances with oxygen partial pressure equality on membrane surfaces and in the shell or lumen, and assuming plug-flow conditions for both gas streams with negligible axial dispersion. The current application of the Tan and Li model [49] to hollow fiber membranes involved a simplified version, assuming a much lower fiber thickness than Lc, allowing the neglect of bulk diffusion resistance. In this case, the surface exchange reaction became the rate-limiting step. The resulting model, expressing the oxygen molar flow rate in the fiber lumen and oxygen permeation flux, can be determined as

(7) $${dN}_{O_{2}}=\frac{k_{r}({{P’}_{O_{2}}}^{\frac{1}{2}}-{{P\prime \prime }_{O_{2}}}^{\frac{1}{2}})}{\frac{{{P’}_{O_{2}}}^{\frac{1}{2}}}{2\pi R_{in}}+\frac{{{P\prime \prime }_{O_{2}}}^{\frac{1}{2}}}{2\pi R_{0}}}dl$$

(8) $$J_{O_{2}}=\frac{k_{r}({{P’}_{O_{2}}}^{\frac{1}{2}}-{{P\prime \prime }_{O_{2}}}^{\frac{1}{2}})}{\frac{{{R_{m}P’}_{O_{2}}}^{\frac{1}{2}}}{R_{in}}+\frac{{{R_{m}P\prime \prime }_{O_{2}}}^{\frac{1}{2}}}{R_{0}}}$$

Tan et al. [60] specified different sets of material balance equations for various operating modes, categorized as Modes 1 to 4. Experimental conditions for oxygen permeation through membranes with different configurations were provided, demonstrating the applicability of Equations (7) and (8) to various hollow fiber geometries. The Tan and Li model’s initial simulation used tubular membranes due to a lack of hollow fiber experimental data. However, the model’s applicability was extended to conventional, asymmetric, and U-shaped hollow fibers, showcasing its versatility in different configurations, particularly highlighting the advantages of U-shaped hollow fibers in co-current purge flow and vacuum operation modes. Thermodynamic and kinetic aspects related to MIEC membrane behavior have been considered, ensuring the model captured both equilibrium and dynamic responses. They verified the accuracy of the MIEC membrane model by comparing it against known data or experimental results, validating the model to ensure it accurately represented the behavior described by the Wagner theory and the Tan and Li model.

The MIEC membrane model was Integrated into the Aspen Plus^®^ simulation environment for system-level simulations. The MIEC membrane model, including details on the Wagner theory, the Tan and Li model, assumptions made, equations, and specific features, has been implemented though Aspen Custom Modeler. The MIEC membrane model is continuously refined and improved based on experimental data obtained from references listed in the bibliography, serving the purpose of designing and sizing the bench-scale testing setup that will be installed at the Polytechnic University of Cartagena. In later stages of the investigation, the developed model will be adjusted and calibrated against the experimental data obtained.

### 3.2. Integrated Heat Recovery System

As was previously discussed, this work is focused on the development of an integrated heat recovery system (IHRS). This approach aims to maximize the utilization of waste heat generated during the co-electrolysis, combining various heat recovery methods and technologies to create a synergistic and efficient solution. Recuperative and regenerative heat recovery systems are two distinct approaches to improving the efficiency of industrial processes by capturing and reusing waste heat. In a recuperative system, heat exchange occurs between two fluid streams, typically a hot process stream and a colder fluid stream. A heat exchanger facilitates this transfer, capturing thermal energy from the hot stream to preheat the incoming cold stream. The fluids flow in separate channels within the heat exchanger, allowing thermal energy transfer and improving overall efficiency. This system is characterized by its straightforward design and operational simplicity, making it cost-effective for various applications.

In contrast, a regenerative system focuses on cyclically storing and releasing thermal energy during different phases of a process. This is accomplished using a heat storage medium or matrix that absorbs heat at high temperatures and releases it during lower-temperature phases. By cyclically changing the flow direction of the working fluid, the system alternates between periods of heat absorption and release, allowing for the recovery and reuse of heat. Regenerative systems tend to be more complex due to the incorporation of valves, storage matrices, and control systems. A regenerative system boasts high efficiency but introduces operational complexities by requiring discontinuous stages to transfer and recover heat through different streams. While the utilization of waste heat is meaningful, challenges emerge when dealing with gas flows, such as air (approximately 10 times the volume of O_2_), which undergoes heating, and O_2_, which requires cooling. The process of air heating demands around 0.3 kWh/Nm_3_ of O_2_ produced, emphasizing the need for highly efficient heat recovery. A concept well-known in Regenerative Thermal Oxidizer (RTO) plants offers a cost-effective solution suitable for very high temperatures using materials like cordierite. In this system, gas flows alternate through a heat storage mass, enabling up to 98% heat recovery with a pressure drop of less than 5 mbar. This innovation ensures a more economical and efficient approach to heat exchange, making it a compelling choice for certain applications [31]. While recuperative systems are simpler and more cost-effective, regenerative systems boast higher efficiency, especially in applications with significant temperature differentials. The choice between the two depends on factors such as the specific industrial process, temperature variations, and economic considerations. Recuperative systems are often preferred for their simplicity and lower initial costs, whereas regenerative systems offer the potential for greater long-term energy savings, particularly in scenarios with larger temperature differentials. The decision ultimately hinges on the unique requirements and characteristics of the industrial application in question [56,57]. Steam has been chosen as carrier due to its flexibility and ease of regulation, making it suitable for applying and distributing the required heat in various working scenarios and system loads. The excess steam generated can be redirected to the steam header and utilized in other processes within the industrial complex. In our case, the superheated steam generated will be employed to preheat and heat the feed streams to the co-electrolyzer. Synergies emerge from the use of steam as a feed for hydrogen production in the stacks [46].

## 4. Results and Discussion

In accordance with the previous section, performance improvements at the cell and system levels of the solid oxide co-electrolyzer can be established through the examination of the thermodynamic process model. This will involve comparing the base case with the case study, presenting the corresponding mass and energy balances. The case study, as well as the stack, system, and plant limits, were established in a previous work by Sánchez-Luján et al. [46]. In the present paper, the benefits derived from the implementation of the integrated oxygen separation and heat recovery system are outlined.

### 4.1. Case Study

In this work, an analysis of an oxygen separation system using MIEC membranes has been conducted, along with the design of a heat recovery system in the anode outlet stream of a co-electrolyzer. The co-electrolyzer produces syngas from captured CO_2_, steam, and renewable electricity, aiming for the generation of e-fuels through Fischer–Tropsch synthesis. The suggested approach relies on a co-electrolysis route to generate syngas. For this analysis, the facility’s production capability was established considering a power supply of 100 MW sourced from renewable energy generation units, encompassing onshore and offshore wind farms, along with PV solar setups. Furthermore, the plant’s capacity is constrained by the electrolyzer’s capabilities (up to 100 MW DC), aligning with the anticipated average capacity of electrolyzers within the EU Hydrogen Valleys [61] and aligning with the capacity of the renewable energy generation system. The design of the unit to produce syngas by co-electrolysis focuses on obtaining a stream of syngas with an H_2_/CO ratio of 2.1 with a minimum content of methane, and free of CO_2_. The unit level conditioning is considered necessary in terms of pressure and temperature for integration with the required PSA for syngas purification prior to the compression stages until the required pressure in the FT reactor is reached [62].

The required heat for conditioning all feeds (water, steam, recycled CO_2_, and H_2_) is generated internally within the facility. In industrial complexes, achieving thermal integration is intricate and challenging, demanding a well-balanced strategy that considers both the production of steam for external use and the utilization of process streams across various blocks or units. The base cases, along with their respective flow sheets and the mass and energy balances, were developed in a previous work of the authors. Findings from the efficiency analysis conducted at both the stack and system levels of the SOEC were presented. When examining the base configuration for the plant’s current capacity and aiming for the required inlet temperature of 800 °C, an additional thermal power input of 2.44 MW is needed. In the context of integration within an industrial complex, all the thermal energy required to achieve the thermoneutral operation of the stack was provided through heat recovery and the thermal integration of process streams from the CPOX reactor. This approach resulted in a 2% increase in performance at all levels for the specified capacity. At the system level, the SOEC efficiency (HHV) reached 84%, supplying syngas and clean CO_2_ at a pressure of 990 kPa [46].

### 4.2. Plant-Level Efficiency Improvement Results

**Figure 5:**
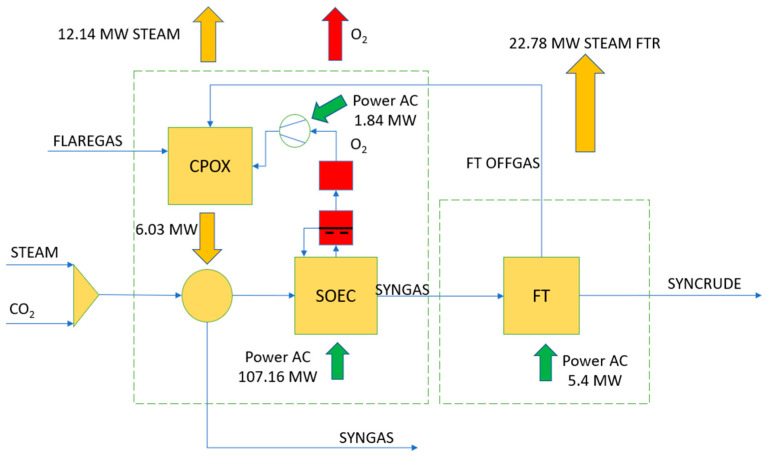
Streams and energy balance process results.

This innovative process configuration allows for a reduction in the equipment required to preheat the sweep gas stream to the co-electrolyzer’s anode. The anode sweep gas stream is recycled after oxygen depletion through MIEC membrane technology, thereby increasing the plant’s steam production capacity by harnessing the heat carried by streams 21 and 22. This results in an additional 4.95 thermal MW of steam production capacity, as depicted in the flow sheet in Figure 4 and Figure 5. This implies a reduction in the carbon footprint of the industrial complex associated with the availability of this additional steam production capacity accounting for emissions savings of 10,000 TPA CO_2_-eq. In terms of the plant’s energy balance, we initially accounted for the oxygen supply through ASU. However, we now only need to factor in the energy cost associated with the vacuum devices and compressors, as there is no requirement for additional thermal energy. This is due to the anode sweep stream maintaining optimal conditions for oxygen separation within the MIEC membrane. For an actual case, the power consumption is quite similar to oxygen generation by ASU. Additional advantages stemming from the implementation of this novel approach and the integrated system for heat recovery and oxygen separation include achieving autonomy in oxygen generation, as the production occurs within the plant’s operational boundaries.

### 4.3. Membrane Area

The primary factors influencing oxygen permeation are the temperature and the O_2_ partial pressure ratio across the membrane. The utilized model in this study allows for the determination of oxygen partial pressure and temperature distribution along the membrane, providing the means to calculate the permeation rate and, consequently, the required membrane area for the desired oxygen flux.

In the four-end mode, a consistently maintained O_2_ partial pressure ratio along the membrane length is ensured. The temperature distribution takes precedence in this mode, exhibiting an increase in the direction of the feed stream, from membrane length 0 to 1. The specific membrane area calculated using the Wagner equation as a shortcut method estimates membrane areas for four-end mode operation between 0.28 m^2^/kWth and 0.22 m^2^/kWth. To enhance the reliability of membrane area estimation, a detailed mathematical model is developed. The model considers flow conditions, allowing the calculation of temperature and oxygen partial pressure along the membrane. This approach calculates discrete, local permeation rates, providing a detailed area estimation. The geometry of the proposed module is based on three-end operation mode of a capillary bunch. The model in the approach of Rautenbach et al. [58] and considers heat transfer within the membrane module. 

In the three-end mode, the membrane temperature remains constant, while the O_2_ partial pressure ratio experiences a gradual decrease along the membrane, reaching a level approximately five times lower compared to the four-end mode. The primary influencing factor on permeation rate in the three-end mode is the distribution of the O_2_ partial pressure ratio. The maximum error between the shortcut method and the detailed model is approximately 2.7%. Given the constant temperature along the membrane tube, any deviation must stem from the calculated average O_2_ partial pressure ratio. Typically, higher efficiencies correlate with increased membrane area. In the three-end concept, this relationship is influenced by the O_2_ partial pressure ratio in the Wagner equation. As efficiencies rise, the permeate pressure increases, leading to reduced energy demand for the vacuum pump and lower O_2_ partial pressure ratios. Specific membrane areas ranging from 0.29 to 0.59 m^2^/kWth have been calculated for the three-end concept. For the case study, the estimated total area for the oxygen separation membrane would be approximately 3680 m^2^.

## 5. Conclusions

The MIEC membranes hold significant potential for applications in oxygen production and as electrodes in solid oxide electrolyzers (SOEC). Despite significant improvements, several technological and research challenges remain unresolved for the commercialization of MIEC membranes in oxygen separation. Advances in computational simulations, modeling, and in situ surface probing techniques are crucial to better understand oxygen permeability mechanisms and performance degradation. These advancements will facilitate the design of materials and engineering approaches to enhance MIEC membrane performance.

Modeling studies play a crucial role as a complement to experimental research on MIEC membranes. Recent developments in model conceptualization have led to the integration of diffusion and surface exchange reactions into a single equation for oxygen permeation flow, such as the Xu–Thomson model and the Tan–Li model. These models have been adapted and applied to various membrane compositions, geometries, and flow configurations. Future developments include more comprehensive multiphysics studies using computational fluid dynamics (CFD).

The results of our research demonstrate the significant potential of integrating MIEC technology with heat recovery systems in solid oxide electrolyzers (Co-SOEC), enabling more efficient utilization of energy resources while minimizing environmental impact. The proposed approach is promising for advancing the commercial viability of Co-SOEC technology and accelerating the transition toward a cleaner and more sustainable energy landscape. The design of experimental tests, once the main causes of failure have been studied and analyzed, is focused on achieving the maximum oxygen flux and the maximum stability and durability of the installation’s membranes.

## Figures and Tables

**Table 1:** Flow sheet MIEC membrane and heat recovery system conditions.

No.	Stream	Temperature (°C)	Pressure (kPa)	No.	Stream	Temperature (°C)	Pressure (kPa)
1	Feed Water	30	180	18	O_2_ Feed	40	1000
2	Feed Steam	250	180	19	Permeate	850	50
3	Steam Mix	142	180	20	O_2_	450	30
4	Steam	550	175	21	O_2_	120	10
5	Hydrogen	350	170	23	Feed Water	30	1000
6	CO_2_	375	170	24	O_2_ + Steam	40	1000
7	Feed Mix	467	170	25	BFW	40	4000
8	Feed Mix	850	160	26	Steam	763.8	3980
9	Syngas	850	150	27	Steam	250.9	3975
10	Syngas	100	125	28	Cooling Water	25	2000
11	Syngas	50	990	29	Cooling Water	40	1995
12	CO_2_	50	220	30	BFW	20	5005
13	Retentate	850	100	31	Steam	263.4	5000
14	Sweep Gas Anode	850	185	32	Steam	270	4995
15	Sweep Gas Anode	850	175	33	Syngas	1066	1000
16	Off-gas Feed	40	1000	34	Syngas	549.2	995
17	Flare-gas Feed	40	1000	35	Syngas	1066	1000

## Data Availability

Data are contained within the article.
